# Editorial: Autophagy in Inflammation Related Diseases

**DOI:** 10.3389/fphar.2022.912487

**Published:** 2022-05-05

**Authors:** Bo-Zong Shao, Pei Wang, Yu Bai

**Affiliations:** ^1^ Department of Gastroenterology, General Hospital of the Chinese People’s Liberation Army, Beijing, China; ^2^ Department of Pharmacology, School of Pharmacy, Navy Medical University/Second Military Medical University, Shanghai, China; ^3^ Department of Gastroenterology, Changhai Hospital, Navy Medical University/Second Military Medical University, Shanghai, China

**Keywords:** autophagy, inflammation, targeted therapy, regulator, pharmacology

Inflammation-related diseases are commonly referred to as a group of diseases induced by the overwhelming triggering of inflammatory responses in the processes of pathogenesis and progression (Zhang et al., 2018). So far, inflammation has been proven to be involved in various kinds of disorders, including cardiovascular diseases (atherosclerosis and myocardial infarction), CNS diseases (cerebral ischemia and stroke), metabolic system (diabetes and obesity), autoimmune diseases (inflammatory bowel diseases and multiple sclerosis) and cancer, etc. (Agirman et al., 2021; Liu et al., 2021). As a result, regulating the inflammatory reaction might serve as a potential and effective strategy in the treatment of such disorders. So far, many mechanisms and signals have been reported to contribute to the regulation of inflammation. Among them, autophagy is one of the most well studied ones (Matsuzawa-Ishimoto et al., 2018; Deretic, 2021). Based on such knowledge, we ran a Research Topic entitled “*Autophagy in Inflammation Related Diseases*,” aiming to provide current knowledge and progression on the application of autophagy in the treatment of inflammation-related diseases. Autophagy is a vital metabolic mechanism in organisms. It is for degrading and recycling long-lived proteins and useless organelles relying on the integration and digestion of lysosomes (Wang et al., 2018). So far, several forms of autophagy have been demonstrated, including three classic forms (microautophagy, macroautophagy and chaperone-mediated autophagy) and several special forms (reticulophagy, mitophagy, etc.) (Wang et al., 2018). The signaling pathways related to autophagy are complex, including AMPK-mTOR signaling and PI3K-Akt signaling, etc. (Wang et al., 2018; Shao et al., 2021). Autophagy has been investigated to regulate inflammation in various kinds of diseases, especially inflammation-related diseases, thus modulating the pathogenesis and progression of such disorders (Shao et al., 2016; Wang et al., 2018; Shao et al., 2021).

In our Research Topic, an article edited by Dr. Shao discussed the role of intestinal macrophage autophagy in inflammatory bowel diseases through reviewing recent related studies (Zheng et al.). The authors discussed the effect of intestinal macrophage autophagy on inflammatory bowel diseases (both ulcerative colitis and Crohn’s disease) in the aspects of autophagy-related gene mutation and modulation of intestinal inflammatory reaction and microbiota. In addition, two kinds of autophagy regulators, namely receptors and receptor regulators and inflammasome regulators have been discussed to illustrate the current application of autophagy in inflammatory bowel diseases. In another article related to inflammatory bowel diseases edited by Dr. Bai, the authors investigated autophagy-related regulators in ulcerative colitis pathogenesis through the analysis of ulcerative colitis patients from GEO database (Qiu et al.). They reported that SERPINA1, an autophagy-related hub gene of active ulcerative colitis, might serve as a novel pharmacological autophagy regulator in ulcerative colitis. SERPINA1 might therefore provide a potential target for the application of small molecular compounds taking advantage of autophagy in the treatment of ulcerative colitis. For another autoimmune disease, rheumatoid arthritis, an article edited by Dr. Shao revealed that tofacitinib, a JAK signaling inhibitor, produced an alleviative effect on rheumatoid arthritis through the modulation of fibroblast-like synoviocytes (Vomero et al.). In skin diseases, an article edited by Dr. Wang summarized the biological features of autophagy and highlighted current findings of the role of autophagy in skin diseases (Klapan et al.). Therapeutic strategies taking advantage of autophagy in the treatment of skin diseases were also introduced and discussed in that review. During the status of infection, a review article edited by Dr. Talero discussed the mechanisms underlying the crosstalk between autophagy and inflammation in the process of infection and further investigated the potential application of such mechanisms in fighting against infection-related damages (Wei et al.). In addition, an article edited by Dr. Wang reported that autophagy contributed to the regulation of coxsackievirus B3-mediated viral myocarditis (Yu et al.). Another article edited by Dr. Talero demonstrated that autophagy played an important role in the treatment of chronic inflammatory diseases through the modulation of CD4^+^ T cell activity (Jeong et al.). Furthermore, the crosstalk between autophagy and inflammation was also revealed by an article edited by Dr. Talero in acute liver injury (Yang et al.).

In conclusion, our Research Topic had reported several latest studies on investigating the role of autophagy in several inflammation-related diseases. We believe that this Research Topic would provide a novel insight in taking advantage of autophagy in the treatment of inflammation-related diseases.
